# Four notable additions to the South African echinoid fauna (Echinodermata, Echinoidea)

**DOI:** 10.3897/zookeys.831.31381

**Published:** 2019-03-18

**Authors:** Zoleka Filander, Yves Samyn, Charles Griffiths

**Affiliations:** 1 Biodiversity and Coastal Research, Oceans and Coast, Department of Environmental Affairs, Cape Town, South Africa Biodiversity and Coastal Research, Oceans and Coast, Department of Environmental Affairs Cape Town South Africa; 2 Zoology Department, Nelson Mandela University, Port Elizabeth, South Africa Nelson Mandela University Port Elizabeth South Africa; 3 Royal Belgian Institute of Natural Sciences, Brussels, Belgium Royal Belgian Institute of Natural Sciences Brussels Belgium; 4 Marine Research Institute, Department of Biological Sciences, University of Cape Town, Rondebosch 7700, South Africa University of Cape Town Rondebosch South Africa

**Keywords:** Biodiversity, new records, taxonomy

## Abstract

Although a comprehensive guide to the South African echinoid fauna was published as recently as 2017, four notable additions to the fauna have emerged since that time and are reported on here. The first South African records for *Histocidarispurpurata* (Thomson, 1872), *Echinothrixdiadema* (Linnaeus, 1758), *Microcyphusrousseaui* L. Agassiz, in Agassiz and Desor 1846, and *Pseudoboletiamaculata* Troschel, 1869 are presented. All four species have previously been recorded from the Atlantic and/or Indian Oceans and their ranges are thus extended southwards here. These additions increase the total number of echinoid species known from South Africa to 74.

## Introduction

The echinoid fauna of South Africa has recently been revised by Filander and Griffiths (2014), who added 19 species to the regional fauna, and by Filander and Griffiths (2017), who provided an identification key and a guide to each of the 70 species then known from the region. Since the publication of that review, four more remarkable additions to the echinoid fauna have been identified and are reported on here. These additions thus increase the number of recorded South African echinoids to 74 species, spread across 29 families.

## Materials and methods

Morphological analysis of the specimens followed the invasive method, which included removal of the primary and secondary spines to expose features of the test. This was done by soaking specimens in a solution of domestic bleach for various time intervals, depending on their size. For the single available specimen of *H.purpurata* the spines were removed from only one half of the test.

Taxa are listed systematically according to Kroh and Smith (2010), which is in line with the classification presented in the World Register of Marine Species (2018), and the scientific name is presented with the author and date of publication. Synonyms are listed in historical order, together with selected literature records under that name. A brief paragraph on the identification features of each species, its previously reported distributional range, and data on the new regional records are also included.

Studied specimens are derived from the following museums:

**SAMC** Iziko South African Museum, Cape Town, South Africa

**RBINS** Royal Belgian Institute of Natural Sciences

**RMCA** Royal Museum for Central Africa, Tervuren, Belgium

## Taxonomic part

### Class Echinoidea Leske, 1778

#### Order Cidaroida Claus, 1880

##### Family Histocidaroidea Lambert, 1900

###### Genus *Histocidaris* Mortensen, 1903

####### Histocidaris purpurata

Taxon classificationAnimaliaCidaroidaHistocidaroidea

(Thomson, 1872)

Fig. 1A, B

Poriocidaris purpurata : Mortensen 1928: 104–107, pl. I, fig. 6, pl. III, figs 3–5 [distribution and synonymy].
Histocidaris purpurata : Clark 1925: 38; Schultz 2011: 872–973, figs.1465–1468; Atkinson et al. 2018: 441.

######## Identification.

Test medium sized (test diameter = 28 mm); round and robust. Marginal series with regular and small tubercles. Interambulacra with distinctively large, crenulate primary tubercles. Areoles slightly deepened. Apical system covered with tubercles, ocular plates exsert. Periproct raised, with plates decreasing in size inwards. Primary spines cylindrical, tapering gently. Oral spines broad, flattened, slightly curved, with serrated edges. Secondary spines flattened, narrowing towards blunt point. Primary spines brown to purplish violet, with white shaft. Secondary spines light brown. Denuded test white.

**Figure 1.**
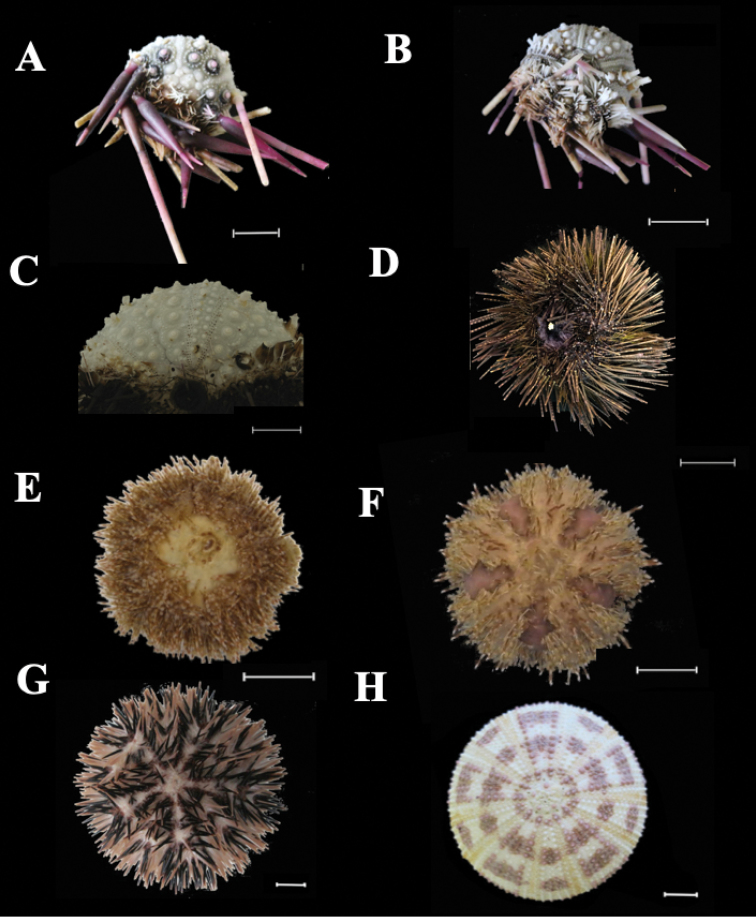
**A–B** (SAMC.A090123, off Mossel Bay): *Histocidarispurpurata*. **A** Aboral view of preserved specimen with partially removed spines **B** Oral view of preserved specimen with partially removed spines **C–D***Echinothrixdiadema***C** (RMCA.2561, Ispingo). Aboral view of partially denuded test **D** (RMCA.2568, Sodwana Bay). Oral view of preserved specimen with spines **E–F** (SAMC.A090124, Sowdana Bay): *Microcyphusrousseaui***E** Oral view of preserved specimen with spines **F** Aboral view of preserved specimen with spines **G–H** (SAMC.A090126, Park Rynie): *Pseudoboletiamaculata***G** Aboral view of preserved specimen with spines **H** Aboral view of preserved denuded test. Scale bars: 2 cm (**A, B, G, H**); 1 cm (**C–F**). All images were edited in GIMP 2.8.22 by Dr Carl Palmer and plate created by Zoleka Filander.

######## Material examined.

SAMC.A090123: one specimen collected by an otter trawl through the South African Observation and Environmental Network/Department of Agriculture and Fisheries long-term offshore invertebrate programme in May 2016 on board the Compass Challenger, depth 570 m. Whole specimen preserved in 70 % ethanol.

######## Habitat.

Muddy habitat.

######## Global distribution.

Previously known only from the Atlantic, Ireland to Canary Islands, and the Caribbean, at 750–1084 m depth (Mortensen 1928; Schultz 2011).

######## South African distribution.

South-east coast of South Africa, off Mossel Bay (35.079°S, 23.603°E).

######## Remarks.

Although Döderlein (1906) previously reported this species in the Indian Ocean (at the Sombrero Channel, Nicobar Islands, at 805 m), Mortensen (1932) disregarded this record on the basis that the specimen was too small and that no adults of this species had been previously collected in the region. The current record therefore represents the first reliable report of this species from the Indian Ocean.

Only one other species, *Histocidariselegans* (A. Agassiz, 1879), belonging to this genus has previously been reported in South African waters and this remains a dubious record, as it lacks locality data (Mortensen 1932; Filander and Griffiths 2017). Nonetheless, *H.purpurata* differs from *H.elegans* both in its distinctive coloration and shape of the primary spines. *Histocidarispurpurata* has a distinctively purple and brown coloration, with thick, cylindrical, and pointed primary spines; whereas *H.elegans* is light brown in colour, with thinner and blunt primary spines.

#### Order Diadematoida Duncan, 1889

##### Family Diadematidae Gray, 1855

###### Genus *Echinothrix* Peters, 1853

####### Echinothrix diadema

Taxon classificationAnimaliaDiadematoidaDiadematidae

(Linnaeus, 1758)

Fig. 1C, D

Garelia cincta : Agassiz 1863: 18–19.
Echinothrix diadema : Mortensen 1940: 290–295, pl. XLIII, figs 1–2, pl. XLV, figs 1–8, pl. XLVI, figs 2–4, pl. XLVII, figs 4, 6–7, pl. XLVIII, fig. 4, pl. LXXI, figs 1, 3 [synonym and description]; Clark and Rowe 1971: 153, fig. 64a [description and distribution]; Samyn and Vanden Berghe 2000: 11 [distribution].

######## Identification.

Test large sized (test diameter = 120 mm). Ambulacra not bulging, with one primary tubercle per three pore-pairs. Interambulacra with distinctively large, perforated, and crenulated primary tubercles, where each plate is surrounded by six smaller tubercles. Areoles slightly deepened. Apical system with insert plates, where gonopores are positioned distally on genital plates. Spines banded, long, and needle-like, with a ridged surface. Denuded test creamy white and reported by Samyn and Vanden Berghe (2000) to be sometimes reddish.

######## Material examined.

RMCA.2561: one specimen collected from the intertidal zone of Isipingo Beach in August 1999; RMCA.2568: one specimen collected by SCUBA diving off 2-Mile Reef in August 1999, at 15 m. All were complete specimens which were fixed and originally preserved in 90–70% ethanol. At present they are preserved dry.

######## Habitat.

Littoral and reef habitats.

######## Global distribution.

Indo-Pacific: Paumotu, Tahiti, Hawaii to Japan, north Australia; to Madagascar, East Africa to Red Sea (Mortensen 1940; Clark and Rowe 1971; Samyn and Vanden Berghe 2000).

######## South African distribution.

East coast of South Africa, south of Durban off Isipingo Beach (30.0036°S, 30.9425°E: approximate co-ordinates), and off 2-Mile Reef, Sodwana Bay (27.5129°S, 32.6862°E: approximate co-ordinates).

######## Remarks.

*Echinothrixdiadema* closely resembles *E.calamaris* (Pallas, 1774), but differs in coloration, patterns of ambulacra, interambulacra, and apical plates. *Echinothrixdiadema* is not reported to have a greenish denuded test, lacks a conspicuous naked interambulacral space, and the ambulacral tubercles increase in size at the ambitus; the apical plates have numerous tubercles (Mortensen 1940).

The current record was not included in previous South African reviews (Filander and Griffiths 2014; Filander and Griffiths 2017) because these publications were based on Iziko South African Museum samples and online accessible samples. Presented here is new material from the Royal Museum for Central Africa, which was not known by the authors at the time (Filander and Griffiths 2014; Filander and Griffiths 2017).

#### Order Camarodonta Jackson, 1912

##### Family Temnopleuridae A. Agassiz, 1872

###### Genus *Microcyphus* L. Agassiz in L. Agassiz & Desor, 1846

####### Microcyphus rousseaui

Taxon classificationAnimaliaCamarodontaTemnopleuridae

L. Agassiz in L. Agassiz & Desor, 1846

Fig. 1E, F

Microcyphus rousseaui L. Agassiz & Desor, 1846: 358, pl. 15.10; Mortensen 1904: 98; Mortensen 1943: 155–159, pl. XIII, figs 18–25, pl. XLVII, figs 18–20, 23–24 [description and synonymy]; Clark and Rowe 1971: 140, 156; Samyn and Vanden Berghe 2000: 6, 13, pl. 1E [distribution]; Schultz 2010: 148, fig. 273 [distribution].

######## Identification.

Test small sized (test diameter = 20 mm), low, hemispherical. Ambulacral pore-pairs arranged in double series, outer series formed by pore-pair of median component, and the inner series by pore-pairs of the upper and lower component of each compound ambulacra plate. Interambulacra partially tuberculated, with sparsely and irregularly arranged same-sized tubercles. Naked part of interambulacra broad and conspicuous. Apical system with apical plates densely covered by tubercles, ocular plates exsert. Periproct covered with numerous plates. Spines of uniform size, reddish-brown with white tips; naked median areas brownish-red, becoming lighter to centre. Denuded test light brown, with darker tuber-covered parts.

######## Material examined.

SAMC.A090124: two specimens collected by SCUBA diving off Leadsman Shoal, Sodwana Bay; SAMC.A090125: one specimen collected by SCUBA diving off Redsands Reef, Sodwana Bay; RBINS I.G. 33199/Ech.132: one specimen collected by SCUBA diving off the 7-Mile Reef, Sodwana Bay. All samples were collected through the Belgian Global Taxonomy Initiative in January 2016, at a 10–23.9 m depth range. All specimens, except for those belonging to sample SAMC.A090124, are complete with spines and preserved in 70 % ethanol.

######## Habitat.

Reef habitat.

######## Global distribution.

Western Indian Ocean: Red Sea, East African coast southwards to Mozambique (Mortensen 1943; Clark and Rowe 1971; Samyn and Vanden Berghe 2001; Schultz 2010)

######## South African distribution.

East coast of South Africa, off Sodwana Bay; off Leadsman Shoal (27.8737°S, 32.6036°E), Redsands Reef (27.7384°S, 32.6298°E), and 7-Mile Reef (27.4515°S, 32.7118°E).

######## Remarks.

Closely resembles *Microcyphusmaculatus* L. Agassiz in L. Agassiz and Desor 1846 from which it differs in the coloration and apical system. *Microcyphusrousseaui* has reddish brown spines and apical plates bearing more than three tubercles, whereas *M.maculata* has light green spines and an apical system bearing fewer than three tubercles per plate (Mortensen 1943).

*Microcyphusrousseaui* differs from *M.rousseauipurpuratus* Mortensen, 1942 in coloration of the spines, the sub-species having purple spines with distinctive white bands (Mortensen 1942).

This is the first South African record of this species and represents a range extension southwards from Mozambique, the southernmost-recorded location. It is also the first record of the genus in the region.

##### Family Toxopneustidae Troschel, 1872

###### Genus *Pseudoboletia* Troschel, 1869

####### Pseudoboletia maculata

Taxon classificationAnimaliaCamarodontaToxopneustidae

Troschel, 1869

Fig. 1G, H

Pseudoboletia maculata Troschel, 1869: 96; Bell 1884: 110, pl. XIII; de Meijere 1904: 286–289, pl. XVII; Clark 1925: 131; Mortensen 1943: 532–534, pl. XLII, figs 4–5, pl. LV, figs 2, 5–6, 16–17, 21 [synonyms and distribution]; Clark and Rowe 1971: 142, 156 [distribution]; Schultz 2010: 264, figs 506–508; Conand et al. 2018: 115.

######## Identification.

Test large sized (70 mm) and low, hemispherical in shape. Ambulacra with pore-pairs arranged in a double series per compound plate, with one larger secondary non-crenulated tubercle outside the pore-pair. Interambulacra with sparsely and irregularly arranged same-sized tubercles, which increase in size towards ambitus. Apical system with smooth apical plates encircled by tubercles, ocular plates I and V appear to be insert. Periproct covered with numerous plates. Spines of uniform size, reddish brown and pinkish white. Denuded test white, with dark brown patches on interambulacra.

######## Material examined.

SAMC.A090126: two specimens collected by Roy Jackson from University of KwaZulu-Natal on an intertidal field trip in August 2015. One specimen is preserved as a naked corona and the other is complete with spines. Both specimens are preserved in 70 % ethanol.

######## Habitat.

Rocky shore.

######## Global distribution.

Indo-West Pacific: Ceylon to Australia, 10–100 m depth (Mortensen 1943; Schultz 2010; Arachchige et al. 2017).

######## South African distribution.

East coast of South Africa, off Park Rynie (30.3187°S, 30.7425°E: approximate co-ordinates).

######## Remarks.

According to our present material, the ocular plates I and V appeared to be insert, which would be consistent with what is observed in other specimens of this species. This is the first South African record, representing a range extension southwards of this species from Madagascar (Clark and Rowe 1971).

## Supplementary Material

XML Treatment for Histocidaris purpurata

XML Treatment for Echinothrix diadema

XML Treatment for Microcyphus rousseaui

XML Treatment for Pseudoboletia maculata

## Supplementary materials

Supplementary material 1 Further additions to the South African echinoid (Echinodermata, Echinoidea) fauna (COL) Data type: species data This dataset is made available under the Open Database License (http://opendatacommons.org/licenses/odbl/1.0/). The Open Database License (ODbL) is a license agreement intended to allow users to freely share, modify, and use this Dataset while maintaining this same freedom for others, provided that the original source and author(s) are credited.Zoleka Filander, Yves Samyn, Charles Griffiths
